# Does Trauma Shape Identity? Exploring the Links Between Lifetime Trauma Exposure and Identity Status in Emerging Adulthood

**DOI:** 10.3389/fpsyg.2020.570644

**Published:** 2020-09-15

**Authors:** Inga Truskauskaite-Kuneviciene, Julia Brailovskaia, Yuka Kamite, Gabija Petrauskaite, Jürgen Margraf, Evaldas Kazlauskas

**Affiliations:** ^1^Center for Psychotraumatology, Vilnius University, Vilnius, Lithuania; ^2^Mental Health Research and Treatment Center, Ruhr University Bochum, Bochum, Germany; ^3^Department of Psychology, Hiroshima University, Hiroshima, Japan

**Keywords:** trauma exposure, identity status, emerging adulthood, sexual trauma, youth

## Abstract

In emerging adulthood, coherent identity plays a protective role against the development of the disturbed psychosocial functioning and is seen as one of the defining characteristics of positive youth development. The factors that shape the identity are still understudied and little quantitative research has addressed, how trauma exposure is linked to emerging adults’ identity. Therefore, the current study aimed to investigate how exposure to traumatic experiences over the lifetime is associated with current identity status among emerging adults in an academic environment and to explore links between different types of traumatic experiences as well as the severity of exposure to trauma and identity statuses. The study sample consisted of 1,614 first-year undergraduate students from Lithuania with the age range of 18–29 years (*M* = 19.09, *SD* = 1.05, 68.28% female). The three distinct identity profiles were identified using the Latent Class Analysis, representing *diffused*, *undifferentiated*, and *coherent* identity statuses. The results provided no clear evidence of links between traumatic experiences and identity status for overall exposure and most types of traumatic events. However, our study concerns the potential importance of severe traumatic experiences, such as sexual trauma, on identity.

## Introduction

The development of a clear, positive, and coherent identity is the main developmental task in adolescence (Erikson, 1968). Positive identity is also conceptualized as an essential defining characteristic of positive youth development (PYD) (Shek et al., 2019; Wiium and Dimitrova, 2019). In the framework of developmental assets, positive identity is seen as one of the four main categories of individual strengths, representing internal assets such as “sense of purpose” and “positive view of personal future” (Benson et al., 2011). In an alternative view of PYD, clear and positive identity is seen as 1 out of 15 developmental constructs, representing the indicators of PYD and, similarly to Eriksonian view of identity, denoting an integrated and coherent sense of self (Catalano et al., 2004). From the perspective of an applied developmental intervention science, fostering coherent identity is seen as one of the avenues for the promotion of PYD (Eichas et al., 2015). Moreover, identity formation is seen as a key process for youth’s well-being and is argued to be closely aligned with thriving and PYD (Arnold, 2017).

The formation of identity continues throughout emerging adulthood, typically, between the graduation of high school and late twenties. Emerging adulthood is a developmental life stage which is characterized by *instability* in terms of personal and career-related relationships, *self-focus*, deriving from relatively few social roles and obligations, *feeling in-between* of child-like adolescence and responsible adulthood, *possibilities and optimism* regarding the future as well as *identity exploration*, that is, trying out various possibilities regarding what kind of person to become and what kind of life to live (Arnett et al., 2014). In emerging adulthood, youth, especially university students, before making any enduring life choices, tend to explore various life alternatives as well as to reflect on diverse available identity choices more than in any other developmental period (Arnett, 2000).

Based on the contemporary process-oriented approach of identity development, which extended Marcia’s (1966) work on identity formation by covering also the concept of identity evaluation, identity development manifests through five main identity processes, namely, exploration in breadth, commitment, exploration in depth, identification with commitment, and ruminative exploration (Luyckx et al., 2008). Exploration in breadth reflects different life alternatives considerations, before making the commitment, and enacting the chosen life path. However, after the choice is made, one can explore their commitments in-depth, actively reflect on them, and gather further information. When the person is satisfied with current enacted commitments, the identification with commitment takes place. If the exploration of possible choices takes dysfunctional form, when the individual tends to doubt over available commitments without being able to choose, this process is called the ruminative exploration which is seen as the only negative process of identity formation (Luyckx et al., 2008).

Marcia (1966) proposed that combinations of different levels in enacted identity processes form unique identity statuses of an individual. The identity statuses characterized by high commitments represent a positive psychological profile with high adjustment and coherent identity; the statuses with lack of commitments and expressed ruminative exploration represent a negative identity profile characterized by a problematic psychosocial functioning and diffused identity (Meeus et al., 1999; Crocetti et al., 2011). The development of coherent identity, in contrast to diffused one, has been shown to be associated with such outcomes as higher self-esteem and life satisfaction, lower anxiety, and depression, as well as lower levels of health risk behaviors (Schwartz et al., 2013). Moreover, it has been recently found that positive identity processes such as commitment making, play an important role in building interpersonal relationships (Kaniušonytë et al., 2019). Therefore, there is an obvious need to understand, how one forms the coherent or diffused identity, and it is particularly important to study the contextual factors contributing to identity development (Beyers and Çok, 2008).

In a previous literature review, it was argued that traumatic experiences may shape the identity development, and the need for more empirical research in this line of research has been emphasized (Berman, 2016). Furthermore, it has been reasoned that traumatic experiences encourage questioning and re-evaluation of current identity commitments and may foster identity diffusion (Berman et al., 2020). However, recent studies of trauma and identity in adolescent samples provided contradictory findings. For example, in the general population, no links were found between stressful life events and identity processes (de Moor et al., 2019), but in the population of adolescent psychiatric patients, sexual and emotional abuse, as well as physical and emotional neglect was associated with diffused and more negative identity (Penner et al., 2019). Also, in the mixed sample of both adolescents and emerging adults, some negative links were found between stressful life events and future career-related commitment (Doeselaar et al., 2018). Nevertheless, evidence from qualitative research suggests that in young adulthood, in the aftermath of trauma, the identity is constructed through the lens of traumatic experiences, that can shape the understanding of oneself in both negative and positive way (Muldoon et al., 2019; Shalka, 2019).

To the best of our knowledge, the association between exposure to traumatic life events and identity statuses in emerging adulthood has never been directly addressed in quantitative research. The estimated prevalence of exposure to potentially traumatic events among college students ranges between 67 and 85% (Grasso et al., 2012). Empirical evidence suggests that in Lithuania the prevalence rate falls within this range with lifetime trauma exposure of 68% among university students (Kvedaraite et al., 2020). Based on previous studies and theoretical conceptualizations, we hypothesized that lifetime trauma exposure could play a role in the enactment of identity in emerging adulthood. Therefore, in the current study, we aimed to investigate how lifetime exposure to traumatic experiences may be associated with identity status among emerging adults. Additionally, we sought to explore links between different types of traumatic experiences as well as the severity of traumatic exposure and identity.

Finally, as a secondary aim, we expected to revalidate previously revealed identity profiles by using the data-driven approach in a specific subgroup of emerging adults from the Lithuanian cultural context. Lithuania is a former Soviet Union country, which was occupied during 1940–1941 and 1944 until 1990. The last three decades of political independence brought major social changes, such as the establishment of democracy and values of the Western countries. With the growing rates of industry and economy, keeping up with larger European countries (Lazutka et al., 2018), Lithuania is becoming increasingly WEIRD (Western, Educated, Industrialized, Rich, and Democratic). However, the majority of current emerging adults’ parents were still raised under the Soviet regime and may still be affected by the post-soviet mentality, which in the interpersonal level might be characterized by prejudice or distrust (Klicperova-Baker and Kostal, 2018).

## Materials and Methods

### Participants and Procedures

In total, 1,710 university students participated in the current cross-sectional study. Data collection took place at one of the biggest universities in Lithuania, which enrolls around 4,000 undergraduates annually. None of the students from the sample were associated with the research team. The research was approved by the Vilnius University Psychological Research Ethics Committee. Data were collected from October to December 2019. The community sampling method was used; all first-year students of the selected university were invited to participate in the study. Students were informed about the study during the integration week events at the university in September 2019 during introductory lectures by study staff. In October 2019, all first-year students were contacted via e-mail and were invited to participate in the study informing when and where the data collection will take place at their Faculty. The research team approached students at the university during regular lecture hours in arrangement with university administration. All students who were present at the lecture at the designated data collection time point were invited to participate and fill-in the online questionnaire and the link to the survey was provided for participants. All participants provided informed consent on the study website prior to data collection. Students who were absent during the data collection at the university were contacted again via e-mail and were provided with a link to the survey with an invitation to take part in the study.

Data from 89 participants were excluded from the analyses because of missing full data in the study measures. Additionally, data from seven participants were excluded because their age was outside of the emerging adulthood age range (>29). The final study sample consisted of 1,614 first-year undergraduate students. The age of study participants ranged from 18 to 29 years (*M* = 19.09, *SD* = 1.05); 68.28% were female. The sociodemographic characteristics of the sample are presented in Table 1.

**TABLE 1:** Sample characteristics.

Variable	*n*	%
**Gender**		
Male	512	31.72
Female	1102	68.28
**Age**		
*M (SD)*	19.09 (1.05)	
Range	18–29	
**Nationality**		
Lithuanian	1530	94.80
Other	84	5.20
**Employment status**		
Employed	265	16.42
Unemployed	1339	82.96
**Monthly family income per person**		
<500 €	1120	69.39
501–1000 €	26	1.61
>1000 €	2	0.12
N/A	455	28.19
**Monthly personal income**		
<400 €	1451	89.90
401–800 €	131	8.12
>800 €	21	1.30
**Relationship status**		
In relationship	695	43.06
Single	911	56.44

**M, mean; SD, standard deviation.**

### Measures

#### Identity Processes

The short form of the *Dimensions of Identity Development Scale* (DIDS; Luyckx et al., 2008: Lithuanian translation Truskauskaite-Kuneviciene, 2017), was used to assess identity processes. The shortened version of the scale includes 12 items, measuring five identity development processes: Exploration in Breadth (two items, e.g., “I am considering a number of different lifestyles that might suit me”), Commitment Making (two items, e.g., “I have decided on the direction I am going to follow in my life”), Exploration in Depth (three items, e.g., “I think about whether the aims I already have for life really suit me”), Identification with Commitment (two items, e.g., “My future plans give me self-confidence”), and Ruminative Exploration (three items, e.g., “I keep wondering which direction my life has to take”). Each item is evaluated on a 5-point Likert scale, ranging from (1) “completely disagree” to (5) “completely agree.” The Lithuanian version of shortened DIDS scale was previously used in other studies and its validity was established (Žukauskienė et al., 2019). Confirmatory Factor Analysis (CFA) of a five-factor model also yielded a good model fit in our study [*χ*^2^ (44) = 359.878, *p* < 0.001, CFI/TLI = 0.952/.928, RMSEA (90% CI) = 0.067 (0.060, 0.073), SRMR = 0.054]. The Exploration in Breadth, Commitment Making, Exploration in Depth, Identification with Commitment, and Ruminative Exploration scales had adequate internal consistency with Cronbach’s alpha coefficients equal to 0.77, 0.85, 0.62, 0.80, and 0.84, respectively.

#### Exposure to Traumatic Experiences

The revised version of *the Life Events Checklist* (LEC-R; Weathers et al., 2013: Lithuanian translation Kazlauskas et al., 2018), was used to determine the lifetime exposure to traumatic experiences among study participants. The LEC-R is a self-report measure that assesses an individual’s exposure for 18 different kinds of potentially traumatic events that happened over the lifetime, ranging from natural disasters to sexual and physical violence. For each event, study participants selected if the event (1) “happened to me,” (2) “witnessed it,” (3) “learned about it,” (4) “not sure,” or (5) “doesn’t apply.” Participants that reported to have experienced (1) or witnessed (2) traumatic event, were considered to be exposed to the traumatic event. Lithuanian translation of LEC-R was used in previously conducted research (Kazlauskas et al., 2018). In the current study, different types of traumatic experiences were classified by the panel of trauma research experts as follows: *disaster* (two items, i.e., natural disaster and/or fire or explosion), *accident* (three items, i.e., transportation accident and/or other serious accident at home/work/during recreational activity and/or exposure to toxic substance), *physical abuse* (two items, i.e., physical assault and/or assault with a weapon), *sexual abuse* (two items, i.e., sexual assault and/or other unwanted sexual experience), *childhood abuse* (two items, i.e., childhood physical abuse and/or childhood sexual abuse), *serious disease and injury* (two items, i.e., life-threatening illness or injury and/or severe human suffering), and *traumatic loss* (three items, i.e., sudden, violent death and/or unexpected death of someone close and/or injury, harm or death you caused to someone else). Based on previous research findings (e.g., Nacak et al., 2017; Michalopoulos et al., 2018), in the current study, experiencing 1 or 2 traumatic events was classified as *non-severe exposure* and experiencing 3 events or more events – as *severe exposure* to traumatic experiences.

### Data Analysis

In the current study, we aimed to investigate whether traumatic lifetime experiences are linked to identity profiles in emerging adulthood. To identify the identity profiles, we used the Latent Class Analysis (LCA) approach (Nylund et al., 2007). We classified the study participants based on the current state of five identity processes, namely, exploration in breadth, commitment, exploration in depth, identification with commitment, and ruminative exploration. Following the LCA approach, we used several criteria to decide on the number of latent classes. First, the Akaike Information Criterion (AIC) and Bayesian Information Criterion (BIC) statistic for a solution with k classes should be lower than for a solution with k–1 classes. Second, a statistically significant *p*-value of the adjusted Lo, Mandel, and Rubin test, which compares improvement in fit between neighboring class solutions after the inclusion of an additional class. Third, we evaluated the substantive meaningfulness of the latent classes (Muthén, 2004). Hence, if a solution with k classes do not have differential substantive meaning, the more parsimonious solution with k–1 classes was chosen. Additionally, in all analyses, we used the Entropy score, with relatively higher values equal or above 0.70 indicative of more accurate classification. When conducting the LCA, we used factor scores (Yang et al., 2010) that were obtained after conducting the Confirmatory Factor Analysis (CFA) of the DIDS scale. The CFA model fit was evaluated by using the Comparative Fit Index (CFI), the Tucker–Lewis Index (TLI), and the Root Mean Square Error of Approximation (RMSEA), following the goodness of fit recommendation provided by Kline (2006); namely, CFI/TLI values higher than 0.90 indicated an acceptable fit and values higher than 0.95 represented a very good fit; RMSEA values below 0.08 indicate of an acceptable fit and values less than 0.05 suggested a good fit. The CFA and LCA analyses were conducted with Mplus 8.2 (Muthén and Muthén, 1998–2017).

To reveal the links between the exposure to traumatic events and identity profiles, we conducted a series of univariate Pearson χ^2^ tests. We first compared the proportions of study participants in identity profiles within trauma exposure vs. the non-exposure group. We identified the participant as being exposed to traumatic experience if at least one out of 18 provided events happened to or was witnessed by the participant. Then, we compared the identity profiles’ proportions within different types of traumatic experiences, namely, disaster, accident, physical abuse, sexual abuse, childhood abuse, serious disease, and injury, traumatic loss. Finally, we compared the proportions in identity profiles within three trauma exposure severity groups, in particular, no exposure, non-severe exposure (1–2 events), and severe exposure (3 events or more). The Pearson χ^2^-tests were conducted with IBM SPSS 24.0.

## Results

### Descriptive Statistics

Means, standard deviations, skewness, kurtosis, and the correlation coefficients of study variables in the sample of Lithuanian first-year university students are presented in Table 2. All study variables, except for the number of traumatic events, were normally distributed, as the coefficients of skewness and kurtosis were within the range of ±2 (Gravetter and Wallnau, 2014). The number of traumatic experiences was not significantly linked to identity processes.

**TABLE 2:** Descriptive statistics and correlations of study variables.

		*N* = 1614		Correlations					
		*M* (*SD*)	*γ*^1^_/_ *γ*^2^	1	2	3	4	5	6
1	Lifetime traumatic events	2.31 (2.19)	1.50/4.27	1					
2	Exploration in breadth	3.72 (0.84)	−0.94/1.25	0.03	1				
3	Commitment making	3.16 (1.05)	−0.29/−0.56	0.03	0.43***	1			
4	Exploration in depth	3.50 (0.77)	−0.46/0.56	0.01	0.25***	−0.09***	1		
5	Identification with commitment	3.11 (0.96)	−0.09/−0.32	−0.04	0.42***	0.71***	−0.04	1	
6	Ruminative exploration	3.36 (0.99)	−0.49/−0.31	0.02	−0.15***	−0.63***	0.36***	−0.58***	1

M, mean; SD, standard deviation; γ^1^, skewness; γ^2^, kurtosis; ***p < 0.001.

### Identity Profiles

**FIGURE 1:**
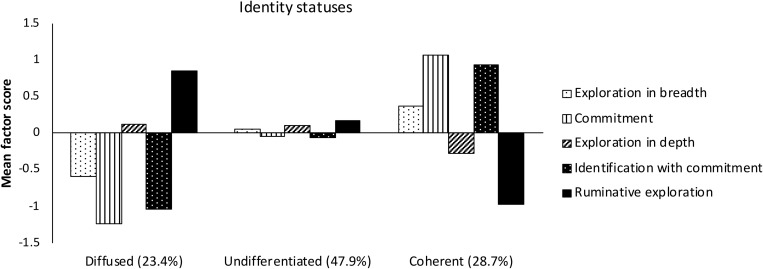
Identity profiles based on factor means of identity processes (*N* = 1614).

The Latent Class Analysis indicated that the three classes solution fitted the data best (see Table 3). Three identified identity profiles were found to be distinguishable in terms of differences in factor means of identity processes (see Figure 1). Two of the distinguished profiles are characterized by exactly opposite combinations of identity processes. The first profile is characterized by low levels of commitment, identification with commitment, exploration in breadth, and high level of ruminative exploration. The second profile, in the opposite, is distinguishable through high levels in both commitments, low level of ruminative exploration, and relatively high exploration in breadth. The third profile seems to represent approximate mean scores in all five dimensions of identity processes. Based on identity literature, the low commitment/high rumination profile was labeled as *diffused* identity status, in contrast, high commitment/low rumination profile was labeled as *coherent* identity status. The profile with no differentiating qualities in terms of identity dimensions was, accordingly, termed as *undifferentiated* identity status.

**TABLE 3:** Model fit indices of latent class analysis.

Solution	Loglikelihood	AIC	BIC	Entropy	LMR-A *p*-value
1 class	−9241.53	18503.05	18556.92	–	–
2 classes	−7862.84	15757.67	15843.86	0.842	<0.001
**3 classes**	−**7083.04**	**14210.09**	**14328.59**	**0.881**	**<0.001**
4 classes	−6734.34	13524.67	13675.49	0.861	0.546

**AIC, Akaike Information Criterion; BIC, Bayesian Information Criterion; LMR-A, Lo-Mendel-Rubin Adjusted Likelihood Ratio Test (LMR-A). Best fitting solution is in bold.**

### Links Between the Exposure to Traumatic Experiences and Identity Profiles

The Pearson χ^2^ tests revealed that the distribution of the study participants across the identity profiles within trauma exposure vs. non-exposure groups did not significantly differ in most cases (see Table 4). Approximately the same proportions of participants in *diffused*, *undifferentiated*, and *coherent* identity profiles groups were found within the groups of exposure vs. non-exposure to overall traumatic experiences, disaster, accident, physical abuse, childhood abuse, serious disease and injury, and traumatic loss. Although overall Pearson χ^2^ test yielded insignificant results, we found that there is a higher proportion of participants with the *coherent* identity profile within no overall trauma exposure group, compared trauma exposure group. Additionally, we found significant effects of sexual abuse exposure on identity profiles; within the sexual abuse exposure group, there was a higher percentage of participants with *diffused* identity profile and a lower percentage of participants with *coherent* identity profile, compared to the proportions within a group without sexual abuse exposure (see Table 4). When comparing the distribution of the study participants across the identity profiles within the severity of trauma exposure groups (no exposure, non-severe exposure, and severe exposure), the overall Pearson χ^2^ test yielded insignificant results. However, we also found that there is a higher proportion of participants with an *coherent* identity profile within no exposure group, compared to non-severe exposure group, when, no differences were found between no exposure and severe exposure groups as well as non-severe and severe exposure groups (see Table 4).

**TABLE 4:** The proportions of participants in identity profile groups within exposure to traumatic experiences groups (*N* = 1614).

Exposure to traumatic experiences (TE) (*n* = no/yes)	Identity statusesh				
	Diffused (*n* = 377)	Undifferentiated (*n* = 773)	Coherent (*n* = 464)		
			
	% within exposure to TE (no/yes) groups (Adj. Res)			*χ^2^*(2)	*p*
Overall (*n* = 366/1248)	21.6/23.9 (0.9)	45.4/48.6 (1.1)	33.1^a^/27.5^b^ (2.1)	4.34	0.114
Disaster (*n* = 1341/273)	24.0/20.1 (1.4)	47.4/50.5 (1.0)	28.6/29.3 (0.2)	1.97	0.373
Accident (*n* = 852/762)	23.8/22.8 (0.5)	47.9/47.9 (0.0)	28.3/29.3 (0.4)	0.30	0.859
Physical abuse (*n* = 1165/449)	23.4/23.2 (0.1)	48.5/46.3 (0.8)	28.1/30.5 (1.0)	1.00	0.606
Sexual abuse (*n* = 1453/161)	22.4^a^/31.7^b^ (2.6)	47.6/50.3 (0.6)	29.9^a^/18.0^b^ (3.2)	12.69	0.002
Childhood abuse (*n* = 1326/288)	22.5/27.1 (1.6)	48.2/46.5 (0.5)	29.3/26.4 (1.0)	2.90	0.235
Serious disease and injury (*n* = 1115/499)	22.2/25.9 (1.6)	48.3/46.9 (0.5)	29.4/27.3 (0.9)	2.63	0.268
Traumatic loss (*n* = 992/622)	23.3/23.5 (0.1)	47.4/48.7 (0.5)	29.3/27.8 (0.7)	0.46	0.796
Severity of traumatic experiences [*n* (no/non-severe/severe) = 366/619/629]	21.6/24.9/22.9 (−0.9; 1.1, −0.4)	45.4/49.9/47.4 (−1.1; 1.3; −0.3)	33.1^a^/25.2^b^/29.7 (2.1; −2.5; 0.7)	7.51	0.111

**Adj. Res = Adjusted standardized residuals. ^*a,b*^Different letters indicate significant differences.**

## Discussion

The current study aimed to investigate whether negative life experiences, in particular, the exposure to traumatic events over the lifetime, are associated with identity status observed during the first college years. Overall, we found no strong evidence of the link between exposure to potentially traumatic experiences and current identity status with some indication that those who were exposed to trauma were less likely to develop coherent identity, compared to those who did not have adverse experiences over their lifetime. Nevertheless, our findings suggest that specific severe traumatic experiences, such as sexual abuse may increase the probability of the development of a more diffused identity.

Overall our findings support the idea that youth may follow positive developmental pathways despite negative life experiences (Oshri et al., 2017). We conceptualize that there may be factors, contributing to positive identity development and mitigating the negative impact of traumatic experiences on identity development. For example, social support as an external resource, and resilience as an inner resource are of great importance when coping with negative life experiences (Thompson et al., 2016). Moreover, positive personality characteristics, such as prosociality, may play an important role in the development of coherent identity (Crocetti et al., 2016) even in face of negative life experiences. Furthermore, the long-term effects of traumatic experiences could result not only in negative outcomes. For example, there is evidence that youth who were exposed to potentially traumatic events with no post-traumatic stress disorder (PTSD) symptoms reported higher levels of perceived social support as well as less avoidance-focused coping (Grasso et al., 2012). Also, there is a great body of literature suggesting that traumatic experiences, despite their adverse content, may also foster some positive changes in life such as post-traumatic growth (Tedeschi and Calhoun, 2004; Joseph et al., 2012), also among youth (Mohr and Rosén, 2017). Moreover, it has been demonstrated that post-traumatic growth is positively linked to such positive identity processes as commitment making, identification with commitment, and exploration in breadth (Žukauskienė et al., 2019). Similarly to the findings in adolescence, the results of our study support the idea that negative life experiences not necessarily result in negative outcomes in terms of identity development (de Moor et al., 2019) and indicate that individual differences may occur when processing traumatic experiences, depending on different factors such as, for example, centrality of event (Berntsen and Rubin, 2006).

However, the results of our study also highlight the importance of making a distinction between different traumatic experiences. Although the exposure to most types of traumatic events was not linked to the current identity profile in our study, we found evidence that sexual trauma was associated with current identity status. In particular, we found that emerging adults, who were exposed to sexual violence or other unwanted sexual experience, were more likely to develop *diffused* identity status, which is represented by expressed negative identity process and less likely to develop *coherent* identity status which is represented by expressed positive identity processes, compared to those who were not exposed to adverse sexual experiences. This finding is in line with previous research, demonstrating that sexual violence is an extremely severe traumatic experience, and victims of sexual violence have a high prevalence of mental health problems, compared to other types of violence (Jakob et al., 2017). Also, sexual violence is an intense negative physical and emotional experience, and has been shown to play an important role in the development of adverse effects on identity (Shalka, 2019). Moreover, experience of sexual abuse is highly associated with shame (Feiring and Taska, 2005) which can undermine the valued social identity (Muldoon et al., 2019) and, in turn, can lead to the violation of the self (Clark, 2014; Muldoon et al., 2016). Our findings indicate that in preventing diffused identity development, it is particularly important to identify the victims of sexual violence and provide timely support for survivors in order to prevent negative developmental outcomes as well as promote the trajectories of positive youth development. Also, the promotion of positive youth development through fostering positive identity for youth survivors of sexual trauma should be integrated into the trauma-informed services, targeted at the empowerment and reduction of symptoms of powerlessness, low self-esteem, and interpersonal difficulties (Bulanda and Byro Johnson, 2016).

In the sample of university freshmen from Lithuania, three distinct identity statuses emerged, when, using the same measure as in other cultures, in particular, North-America (Schwartz et al., 2011) and Italy (Crocetti et al., 2011), six identity statuses were identified. The status that we labeled as *diffused* identity, in other studies represented the *diffused diffusion* identity profile. The *coherent* identity status that we found in our study is closest to the *foreclosure* identity profile found in other studies. However, for *coherent* identity, alongside high levels of commitments, we found relatively higher levels of exploration in breadth, especially in comparison to exploration in depth, when *foreclosure* is characterized by similarly lower levels of both explorations. Our findings suggest that identity statuses may vary across cultures and specific samples. We believe, our findings regarding the number and characteristics of the identity profiles may be also attributable to the features of our sample. Possibly, emerging adults who just started studying at the university may not yet demonstrate *foreclosure*, as many changes occur in their life and they still showed relatively higher levels of exploration. Also, they may not yet have enough space for *achievement* (with high levels of both commitments and both explorations), as they need to concentrate on current newly emerged tasks instead of being involved in exploring different directions in life or expansively reflecting on current ones. Moreover, almost half of the Lithuanian emerging adults’ sample in our study demonstrated the undifferentiated identity profile, being the largest proportion among three identified profiles, when, for example, in Italy, one out of four students was characterized by this identity status (Crocetti et al., 2011). These results may be specific to the Lithuanian cultural context and may indicate that youth in Lithuania reflect country-level challenges regarding the enactment of the identity questions (King and McNabb, 2014).

Although we did not find clear evidence that traumatic experiences may shape identity formation, the links between trauma and identity can still be there. It has been argued that the relationship between identity and trauma is complex and bidirectional. Not only trauma may affect identity development, but also identity style may influence how the person deals with adverse life experiences (Berman, 2016; Berman et al., 2020). The cross-sectional design of this study did not enable us to explore the development of identity. Moreover, we were unable to control for possible gender and socioeconomic background effects on links between trauma exposure and identity style. To address the question of the bidirectionality and complexity of the links between trauma and identity, the longitudinal study design is needed. The other limitation of our study is the sampling method used. The study data is from the largest university in Lithuania; however, this university also has the highest rating in the country, therefore, the results may be more attributable to the students that showed higher education achievement in high school. Also, our sample did not include emerging adults who were not enrolled in secondary education. Finally, as it is often the case in studies with university students (Schwartz et al., 2011), our sample is disproportionally female. Therefore, to further explore, whether traumatic experiences play the role in identity development for emerging adults, it would be worthwhile to replicate the current findings with gender-balanced non-student samples.

Despite these limitations, the results of our study in a large sample of emerging adults from the understudied cultural context inform both trauma and identity-related research that exposure to traumatic experiences may not be among critically important factors when linking the developmental context and the formation of coherent identity. However, our study also informs that severe traumatic experiences, such as sexual trauma, could have a negative effect on identity and should be explored in further studies. Thus, for now, we can conclude that not any exposure to traumatic events, but rather specific difficult traumatic experiences may undermine positive youth development by shaping how the young adults from the educational context address identity questions.

## Data Availability Statement

The raw data supporting the conclusions of this article will be made available by the authors, without undue reservation, to any qualified researcher.

## Ethics Statement

The study was reviewed and approved by the Vilnius University Psychological Research Ethics Committee. The participants provided their written informed consent to participate in this study.

## Author Contributions

IT-K: research planning, data collection, data analysis, writing up the first draft, and contribution to obtaining funding. JB: research planning and comments and improvements of the first draft. YK: comments and improvements of the first draft and contribution to obtaining funding. GP: data collection and contribution to the “Materials and Methods” section. JM: research planning and supervision. EK: research planning, data analytic plan, comments and improvements of the first draft, supervision, and contribution to obtaining funding. All authors contributed to the article and approved the submitted version.

## Conflict of Interest

The authors declare that the research was conducted in the absence of any commercial or financial relationships that could be construed as a potential conflict of interest.
